# The effect of BCG on iron metabolism in the early neonatal period: A controlled trial in Gambian neonates

**DOI:** 10.1016/j.vaccine.2015.04.087

**Published:** 2015-06-12

**Authors:** Sarah Prentice, Momodou W. Jallow, Andrew M. Prentice, MRC-International Nutrition Group

**Affiliations:** a Department of Clinical Research London School of Hygiene and Tropical Medicine, Keppel Street, London WC1E 7HT, UK; b MRC International Nutrition Group, MRC Keneba, The Gambia

**Keywords:** ISRCTN93854442, BCG, Iron, Hepcidin, Heterologous effects, Neonate, BCG, Bacillus Calmette-Guerin, EDTA, ethylenediaminetetraacetic acid, ELISA, enzyme linked immunosorbant assay, HBV, Hepatitis B Vaccine, IL-6, interleukin 6, OPV, oral polio vaccine, TSAT, transferrin saturation

## Abstract

Bacillus Calmette-Guerin (BCG) vaccination has been reported to protect neonates from non-tuberculous pathogens, but no biological mechanism to explain such effects is known. We hypothesised that BCG produces broad-spectrum anti-microbial protection via a hepcidin-mediated hypoferraemia, limiting iron availability for pathogens.

To test this we conducted a trial in 120 Gambian neonates comparing iron status in the first 5-days of life after allocation to: (1) All routine vaccinations at birth (BCG/Oral Polio Vaccine (OPV)/Hepatitis B Vaccine (HBV)); (2) BCG delayed until after the study period (at day 5); and (3) All routine vaccinations delayed until after the study period.

Vaccine regime at birth did not significantly impact on any measured parameter of iron metabolism. However, the ability to detect an effect of BCG on iron metabolism may have been limited by short follow-up time and high activation of the inflammatory-iron axis in the study population.

## 1 Background

The possibility that BCG vaccination might protect neonates against non-tuberculous infections has been suggested by two randomised controlled trials [1,2] and numerous epidemiological studies [3–7]. However, the theory has failed to gain acceptance, partly due to the lack of a putative biological mechanism to explain such effects. The randomised trials indicated that protection was strongest within 3 days post-vaccination thus implicating an effect on innate immunity [2]. We theorised that BCG might mediate its heterologous effects by stimulating an iron-withholding response, as part of an acute phase reaction to vaccination.

Iron is critical for the growth and virulence of the majority of human pathogens [8]. The acute phase response produces a rapid reduction in serum iron limiting its availability for pathogens. This hypoferraemia is thought to be primarily orchestrated by IL-6 (and possibly other inflammatory cytokines) up-regulating hepcidin in the liver. The iron-regulatory hormone hepcidin acts on macrophages and enterocytes to internalise the transmembrane iron-transporter protein ferroportin. This sequesters circulating iron within macrophages and reduces enteric absorption of dietary iron.

The kinetics of iron metabolism in the early neonatal period are poorly described, but it is believed to be a period of high iron flux. Fetal red cell mass is higher than post-natally [9], with excess erythrocytes broken down in the first few days following birth. Difficulties metabolising the haem component of haemoglobin are commonly seen in neonates, in the form of jaundice. High iron loads may contribute to the enhanced risk of infections that occur during the neonatal period, exemplified by the 20-fold increased risk of *Escherichia coli* sepsis that occurred in Polynesian infants following provision of iron dextran at birth [10]. Thus, reduction of serum iron as an innate immune strategy to limit the growth of pathogens may be particularly beneficial in the neonatal period.

The effects of BCG, and other vaccines, on the inflammatory-iron pathway in humans are unknown. Several lines of evidence, however, suggest that impacts on this pathway do occur: (1) BCG is a strong inducer of IL-6 [11] and other innate cytokines [12] *in-vivo*; (2) live-vaccinations similar to BCG produce strong up-regulation of hepcidin in fish [13]; and (3) BCG in guinea-pigs leads to a rapid bacteriostatic hypoferraemia [14].

We therefore conducted a proof-of-principal controlled trial in Gambian neonates to investigate the impact of BCG, and other vaccines received at birth, on iron metabolism in the first five days of life.

## 2 Methods

80 healthy Gambian neonates were randomly allocated to receive BCG (Danish Strain 1331, Batch 11023B, 0.05 ml intra-dermally into the left deltoid) either at birth, or after completion of study procedures at five days old. All other routine immunisations (Oral Polio Vaccine (OPV)) and Hepatitis B Vaccine (HBV) were given at birth as normal. A data manager not directly involved in the study, conducted randomisation using Microsoft Access, upon delivery of an eligible infant. Blocked randomisation using blocks of six with a 1:1 allocation ratio was used. Due to concerns regarding the potential confounding influence of OPV and HBV at birth, a third non-randomised group of 40 infants was subsequently recruited and received all vaccinations after completion of study procedures at five days of age. Recruitment ran from May 2013 until February 2014, with the first two, randomised groups, recruited during both rainy and dry seasons, and the third non-randomised group recruited during the dry season.

All participants had a 2 ml baseline venous blood sample taken within 24 h of delivery, prior to receipt of any vaccinations, and a further 2 ml venous blood sample taken either 24–48 or 72–96 h post-intervention. Blood was collected directly into microtainers (Becton–Dickson: 0.5 ml collected into EDTA containing tubes, 1.5 ml into lithium–heparin containing tubes) from the dorsum of the hand. Full blood counts were assessed from EDTA blood using the automated Medonic analyser. Lithium–heparinised blood was centrifuged for 4 min at 3600 g within 4 h of collection and the plasma stored at −70 ˚C until analysis. Iron parameters were measured using the automated Cobas Integra 400 plus (Roche Diagnostics). Plasma hepcidin was measured in duplicate, using a 1:20 dilution by competitive ELISA (Bachem-25, USA) with detection range 0.02–25 ng/ml. Plasma IL-6 was measured in duplicate using a 1:2 dilution by competitive ELISA (BD OptEIA, Oxford, UK), with detection range 0.49–250 pg/ml. Samples with readings outside the linear portion of the curve were re-run at alternative dilutions. Values below the limit of detection were imputed using limit of detection/√2. Any samples with an intra-assay co-efficient of variance >15% were re-analysed.

Demographic, birth details and anthropometry were collected at enrolment. Due to the rural nature of the study site, all births were vaginal. Deliveries and follow-up visits were conducted at the participant's home.

Full informed consent was obtained from mothers antenatally by a trained midwife. Inclusion criteria were (1) Consenting mother (2) Residence within the study area. Exclusion criteria were (1) Infant weighing <2000 g (2) Maternal HIV or TB (3) TB contact in the home (4) complicated delivery (5) major congenital anomaly (6) infant unwell as judged by a doctor or a midwife. The Consort flow diagram for the study can be found as supplementary material.

Clinical investigators and mothers were not blinded to intervention allocation due to lack of feasibility (BCG produces a visible reaction) and for safety, so that any mothers would be aware of the vaccination status of the child. Laboratory investigators were blinded to intervention allocation, with assays conducted by anonymous study number. Data were analysed using Stata Version 11.0. Categorical variables were compared using the chi-squared test and continuous variables by one-way ANOVA. Hepcidin and IL-6 results were not normally distributed and were log-transformed prior to comparison. Intervention allocation code was not broken until the data were cleaned and locked.

As this study was a small proof-of-principal trial, with short follow-up and no clinical endpoints, no data safety monitoring board was appointed. Safety data were monitored in real time by clinical investigators who were not blinded to intervention allocation. There was no significant difference in incidence of serious adverse events by intervention allocation group (see Table 1).

Ethical approval was obtained from the joint Gambia Government/MRC Unit The Gambia ethics committee (Ref: SCC1325) and the London School of Hygiene and Tropical Medicine ethics committee (Ref: 012-045). This trial was conducted according to the principles of the Declaration of Helsinki.

## 3 Results

Baseline demographic variables were balanced amongst the three intervention groups (Table 1), suggesting that adequate randomisation occurred and that the third, non-randomised arm, was comparable.

**Fig. 1:**
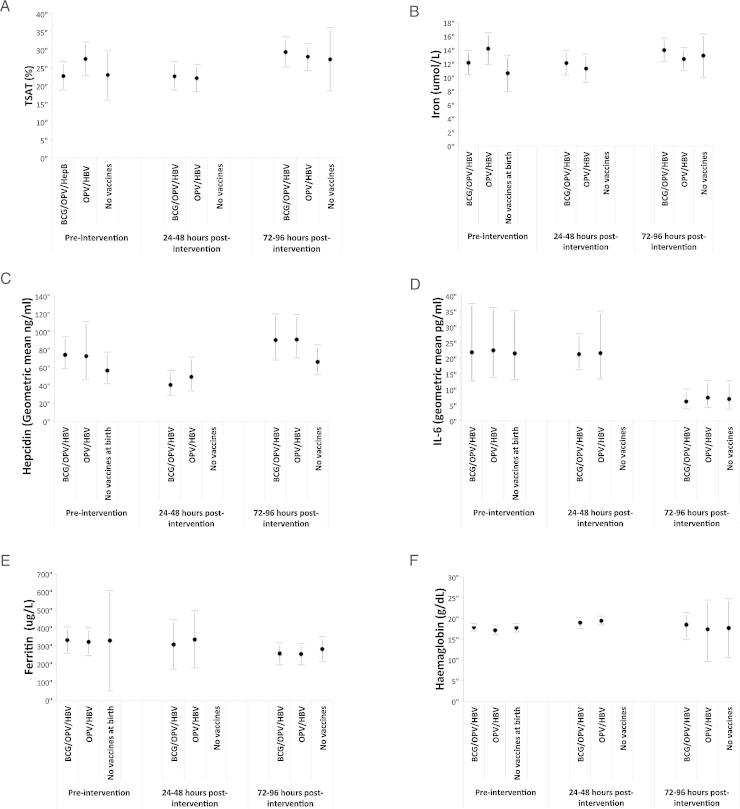
Iron parameters (means ± 95% confidence intervals) by intervention group and time post-intervention.

As shown in Fig. 1, there was no significant impact of BCG or other routine immunisations received at birth on any measured parameters of the inflammatory-iron axis at either 24–48 h or 72–96 h post-intervention. No significant differences were found when comparing (1) intervention groups at each blood sampling point (Table 2), (2) within-infant changes to parameters over time by intervention group and (3) infants receiving any vaccines at birth (groups 1 and (2) with vaccination naïve infants (group 3) (data not shown, all *p*-values > 0.05). The hepcidin levels in group 3 (recruited separately in the dry season) showed a trend toward being lower at all time-points. However this finding was not significant and was not reflected by higher iron or TSAT levels. It is thus unlikely to represent a true difference.

As previous trials reported more significant effects of BCG in male infants results were also analysed by gender (Table 2). In general no differences in the impact of vaccine timing on parameters by gender was found. However, IL-6 was significantly higher in male infants receiving BCG at birth than delayed (*p* = 0.02), and hepcidin which was significantly lower in girls who had received all vaccines delayed (*p* = 0.004). As these findings were not reflected in changes to any other parameters of the inflammatory-iron axis, they may reflect multiple testing artefacts.

## 4 Discussion

This study found no evidence that BCG or other routine immunisations at birth impact significantly on iron metabolism. However, we may have failed to identify an inherent ability for vaccinations to stimulate the inflammatory-iron pathway for a number of reasons:

First, BCG is a slowly replicating live-organism and may take time to reach a level in the body able to stimulate a systemic response. The later time-point of 72–96 h post-vaccination may have been too early to identify any impact of BCG on iron metabolism.

Second, mean IL-6, hepcidin and ferritin levels in these neonates were high, with IL-6 initially 10–20 fold higher [15], hepcidin 1.5–2 fold higher [16] and ferritin 5–10 fold higher [17] than reported circulating levels in older children. Correspondingly TSAT and iron levels were at the lower end of the normal range, approximately 50% lower than previously reported ranges from cord blood [18]. This suggests that the inflammatory-iron axis, whether mediated by hepcidin-dependent or independent pathways [19] was already stimulated in all of our study participants, perhaps due to acute inflammation precipitated by the birth process [20]. If the axis is already maximally stimulated in these infants any additional impact of BCG or other vaccines would not have been detectable. The non-specific effects of BCG are reportedly highest in low birth-weight/premature infants. It may be that stimulation of the inflammatory-iron axis at birth is blunted in this population and is enhanced by immunisations. Thus, impacts on the iron-inflammatory axis cannot be ruled out as a potential biological mechanism to explain the non-specific effects of BCG in such babies.

To fully understand whether BCG and other routine immunisations have an impact on iron metabolism, similar studies in premature neonates and older infants, from different geographical regions and with longer blood sampling time points, are necessary.

## Figures and Tables

**Table 1:** Population characteristics by intervention group.

	Group 1	Group 2	Group 3	*p*-Value^c^
	BCG/OPV/HBV	OPV/HBV	No vaccines	
	*n* = 40	*n* = 40	*n* = 40	
Gender (male, %)	51.2	48.7	47.5	0.94
Gestational age (weeks)	38.2	38.0	38.1	0.89
Birth weight (g)	3065	3069	3045	0.71
Length (cm)	50.8	50.5	50.7	0.91
Head circumference (cm)	34.4	34.1	34.1	0.48
Parity	3.2	3.6	4.3	0.48
Maternal iron supplementation	95.1%	100%	97.5%	0.38
Timing of pre-intervention blood sample (hours)	6.85	5.92	7.69	0.29
Admissions to hospital during study period^a^	1	2	1	1.0
Deaths during study period^b^	0	0	1	0.33

a All admissions were for presumed neonatal sepsis. All infants received antibiotics and improved within 48 h. They were discharged when blood cultures were negative.

b One study participant died at home between the first and second study visits, cause of death unknown.

c Categorical variables were compared using the Chi-squared test. Continuous variables were compared using one-way ANOVA.

**Table 2:** Comparison of mean iron metabolism pathway parameters by intervention group and time post-intervention.

	Pre-intervention (<24 h of age)				24–48 h post-intervention				72–96 h post-intervention			
	Group 1	Group 2	Group 3	*p*-Value^b^	Group 1	Group 2	Group 3	*p*-Value	Group 1	Group 2	Group 3	*p*-Value
	*n* = 39^a^	*n* = 37	*n* = 35		*n* = 17	*n* = 15	*n* = 0^c^		*n* = 20	*n* = 20	*n* = 25	
Iron (μmol/L)	12.2	14.2	10.6	0.08	12.0	11.3	–	0.65	14.0	12.7	13.2	0.72
Male	*11.5*	*13.4*	*11.4*	*0.55*	*11.3*	*10.5*		*0.49*	*14.0*	*12.0*	*12.5*	*0.55*
Female	*12.9*	*15.3*	*9.6*	*0.08*	*12.7*	*12.8*		*0.98*	*14.0*	*13.1*	*13.9*	*0.97*
TSAT (%)	22.8	27.5	23.1	0.37	22.5	22.2	–	0.89	29.4	28.1	27.4	0.88
Male	*21.1*	*26.4*	*22.9*	*0.46*	*22.8*	*21.2*		*0.72*	*30.5*	*27.2*	*24.3*	*0.30*
Female	*25.3*	*28.9*	*23.4*	*0.71*	*22.0*	*24.1*		*0.79*	*28.2*	*28.9*	*30.9*	*0.93*
Hepcidin (ng/ml)^d^	74.5	72.9	56.9	0.52	40.9	49.8	–	0.41	91.0	91.7	66.5	0.32
Male	*76.7*	*100.2*	*63.1*	*0.74*	*35.1*	*49.4*		*0.51*	*80.9*	*85.1*	*89.2*	*0.87*
Female	*72.0*	*54.0*	*51.6*	*0.33*	*49.7*	*50.3*		*0.86*	*101.3*	*98.8*	*48.4*	*0.004*
IL-6 (pg/ml)^d^	22.0	22.6	21.6	0.71	21.4	21.7	–	0.12	6.3	7.5	7.1	0.90
Male	*30.6*	*28.1*	*21.3*	*0.62*	*24.5*	*18.1*		*0.02*	*5.8*	*10.3*	*7.6*	*0.54*
Female	*15.8*	*17.4*	*22.0*	*0.44*	*16.7*	*26.1*		*0.39*	*6.8*	*5.3*	*6.6*	*0.94*
Ferritin (μg/L)	333.6	324.2	330.9	0.99	308.7	337.3	–	0.43	259.1	256.3	283.5	0.75
Male	*287.0*	*282.8*	*367.0*	*0.85*	*250.4*	*293.9*		*0.54*	*227.9*	*235.1*	*285.0*	*0.53*
Female	*393.6*	*371.5*	*240.6*	*0.27*	*396.2*	*380.7*		*0.92*	*304.3*	*268.2*	*281.6*	*0.86*
Haemoglobin (g/dL)	17.9	17.2	17.8	0.47	18.8	19.5	–	0.45	18.5	17.4	17.7	0.65
Male	*17.4*	*16.8*	*17.2*	*0.72*	*18.0*	*19.5*		*0.10*	*18.3*	*15.7*	*17.5*	*0.15*
Female	*18.4*	*17.7*	*18.5*	*0.70*	*20.0*	*19.4*		*0.60*	*18.6*	*19.5*	*17.9*	*0.68*

a Number for each group is the maximum number of blood samples available. Not all parameters were available for all samples due to volume constraints.

b One-way ANOVA.

c Infants in group 3 were only sampled at the 72–96 h sampling time-point.

d Geometric means.
