# Supplementary material for: The effect of BCG on iron metabolism in the early neonatal period: A controlled trial in Gambian neonates
Source: Vaccine. 2015 Jun 12;33(26):2963–7. doi: 10.1016/j.vaccine.2015.04.087 (PMC4503799; doi:10.1016/j.vaccine.2015.04.087)
Supplement: Supplementary file 1 [file mmc1.docx]

**Consort Study Flow Diagram**
